# Robust Synthesis of Ciprofloxacin-Capped Metallic Nanoparticles and Their Urease Inhibitory Assay

**DOI:** 10.3390/molecules21040411

**Published:** 2016-03-25

**Authors:** Muhammad Nisar, Shujaat Ali Khan, Mughal Qayum, Ajmal Khan, Umar Farooq, Hawa Z.E. Jaafar, Muhammad Zia-Ul-Haq, Rashid Ali

**Affiliations:** 1 Office of Research Innovation and Commercialization, University of Peshawar, Peshawar-25120, Pakistan; 2 Institute of Chemical Sciences, University of Peshawar, Peshawar 25120, Pakistan; 3 Department of Pharmacy, Kohat University of Science and Technology, Kohat 26000, Pakistan; mu_afridii@yahoo.com; 4 Department of Chemistry, COMSATS Institute of Information Technology, Abbottabad 22060, Pakistan; umarf@ciit.net.pk (A.K.); ajmalkhan@ciit.net.pk (U.F.); 5 Department of Crop Science, Faculty of Agriculture, 43400 UPM Serdang, Selangor, Malaysia; 6 Office of Research, Innovation and Commercialization, Lahore College for Women University, Lahore 54600, Pakistan; 7 Department of Physics, University of Malakand, Khyber Pakhtunkhwa 18800, Pakistan; rashidalikhan776@gmail.com

**Keywords:** metallic nanoparticles, ciprofloxacin, urease enzyme inhibition, antibacterial activity

## Abstract

The fluoroquinolone antibacterial drug ciprofloxacin (cip) has been used to cap metallic (silver and gold) nanoparticles by a robust one pot synthetic method under optimized conditions, using NaBH_4_ as a mild reducing agent. Metallic nanoparticles (MNPs) showed constancy against variations in pH, table salt (NaCl) solution, and heat. Capping with metal ions (Ag/Au-cip) has significant implications for the solubility, pharmacokinetics and bioavailability of fluoroquinolone molecules. The metallic nanoparticles were characterized by several techniques such as ultraviolet visible spectroscopy (UV), atomic force microscopy (AFM), Fourier transform infrared spectroscopy (FTIR), scanning electron microscopy (SEM) and energy dispersive X-ray (EDX) methods. The nanoparticles synthesized using silver and gold were subjected to energy dispersive X-ray tests in order to show their metallic composition. The NH moiety of the piperazine group capped the Ag/Au surfaces, as revealed by spectroscopic studies. The synthesized nanoparticles were also assessed for urease inhibition potential. Fascinatingly, both Ag-cip and Au-cip NPs exhibited significant urease enzyme inhibitory potential, with IC_50_ = 1.181 ± 0.02 µg/mL and 52.55 ± 2.3 µg/mL, compared to ciprofloxacin (IC_50_ = 82.95 ± 1.62 µg/mL). MNPs also exhibited significant antibacterial activity against selected bacterial strains.

## 1. Introduction

In recent decades, the use of nanomaterials, especially metal nanoconjugates, has spread in biomedicinal research. The following factors play a key role when it comes to the use of nanomaterials in different diagnostic as well as therapeutic techniques: (i) nano size; (ii) higher surface area in comparison to the total volume of nanoparticles; (iii) good affinity for live cells; (iv) stable nature over a considerable temperature range. The special optical properties associated with nanoparticles make them suitable for imaging applications as immense data is available featuring diversity in size and other characteristics. This diversity is justified as metallic nanoparticles have a tendency to interact with their neighbours producing new nanoparticles with diverse features. The literature reveals plenty of data regarding iron, titanium oxide and gold nanoparticles. [1]. Metallic NPs exhibit entirely new or better properties, which are fairly unique from those of larger particles. As compared to the larger particles of the bulk material, they possess specific characteristics such as shape, size, surface morphology and distribution [2]. The low cytotoxic nature of gold and silver as well as their less reactive characteristics make them ideal for their applications in gene therapy and drug delivery [3].

Nanotechnology is an emerging field having an outstanding impact in all spheres of human life [4]. Although size of nanoparticles decides their applications in the drug industry, size still has its pros and cons, as unwanted penetration of nanoparticles can cause significant damage. This reduces the specificity of nanoparticles towards a certain group of cells. This problem has been resolved to some extent by using certain ligand molecules along with nanoparticles to help the nanoparticles in specified drug delivery at the desired place. Despite these demerits, nanoparticles have been the focus of researchers during the past two decades, which indicates their immense importance [5,6,7,8].

Different methods have been reported so far for the synthesis of metallic nanoparticles like chemical methods and biological methods. In all these methods changing the thermodynamic or kinetic variables as well as physical factors allows one to vary the nanoparticle features. Devising ways to make gold colloids was the center of attention for several scientists over a long period of time [9,10]; the fastest was the reduction of gold salts [9]. Silver is the second most important metal when it comes to nanoparticle synthesis. Several schemes have been established to synthesize silver nanoparticles as well. The smaller size of silver nanoparticles makes it easy for them to form aggregates; these aggregate formations can be avoided with the use of several stabilizers that have the capacity to stick to the nanoparticles, for instance, certain polymers and polyelectrolytes [11].

Recently the coupling of nanoparticles with drugs has been shown to produce a marked increase in the activity of the drugs, like antibiotics which when combined with nanoparticles display increased killing of bacteria [12,13,14,15,16,17,18,19,20,21,22,23,24,25,26,27,28]. Metallic NPs reveal excellent antimicrobial activity against various pathogens such as fungi, bacteria and viruses and can play a key role in nanomedicine [29]. Urease belongs to the urea amido-hydrolase enzyme class. It speeds up the conversion of urea into ammonia and carbamate by hydrolysis. Carbonic acid is formed at physiological pH by the hydrolysis of carbamate. Urease is well known for the pathologies induced by *Helicobacter pylori* [30]. Urease facilitates the growth of *H. pyroli* with in the stomach, thereby facilitating the cancer progression. The involvement of urease in urolithiasis has been proven in literature; it has roles in arthritis as well as acute pyelonephritis as well [31,32].

Urea is the primary nitrogen-containing waste product in certain living organisms; it is rapidly treated within the body by microorganisms. The plant kingdom, animal kingdom and fungi contain members which have urease activities going on in their bodies [33]. In farming, urease activity is related with several environmental and financial threats. The rapid degradation of urea caused by ureolytic bacteria in the soil increases soil ammonia levels. This urea degradation and high levels of ammonia within the soil are hazardous for the plants [34]. In the seed germination process, urease exhibits a dynamic role in the digestion of nitrogen [35,36].

Finding new inhibitors for urease has been an area of interest for scientists in the present era [37]. Quinolones comprise a group of well-known bactericides; they are considered as good antibacterial agents and quinolones have been in phase I and more advanced clinical trials since the 1970s [38,39,40]. Quinolones contain a 4-oxo-1,4-dihydroquinoline skeleton [41,42]. They positively stop the replication of genetic material and are can cure numerous infections [43]. In contrast to first- generation quinolones (cinoxacin, nalidixic acid), second generation ones (enoxacin, ciprofloxacin, norfloxacin, and ofloxacin), show activities against Gram-negative and Gram-positive bacteria and also against certain pathogens [44,45]. Ciprofloxacin has revealed extended activity against Gram negative and Gram positive bacteria as well as activity against anaerobes and atypical bacteria. It performs a dual action, inhibiting DNA gyrase and topoisomerase IV, which slows the development of resistance [46]. As compared to the free drug molecules, the pharmacokinetics and therapeutic efficacy of drugs can be substantially enhanced by loading drugs onto the surface of nanoparticles via physical encapsulation, chemical conjugation or surface adsorption [47].

The current studies focus on a facile, robust and simple synthesis of noble metal nanoparticles (Ag/Au NPs) by the Tarkevisch method using sodium borohydride as a mild reducing agent and their biological activity [48,49]. Ciprofloxacin, a fluoroquinolone antibiotic was used for stabilizing and capping of Ag/Au-cip NPs. In particular the amine moiety of ciprofloxacin was responsible for capping the silver/gold nanoparticles. The urease inhibitory assay of ciprofloxacin conjugated with Ag/Au NPs was also reported for the first time.

## 2. Results and Discussions

**Figure 1:**
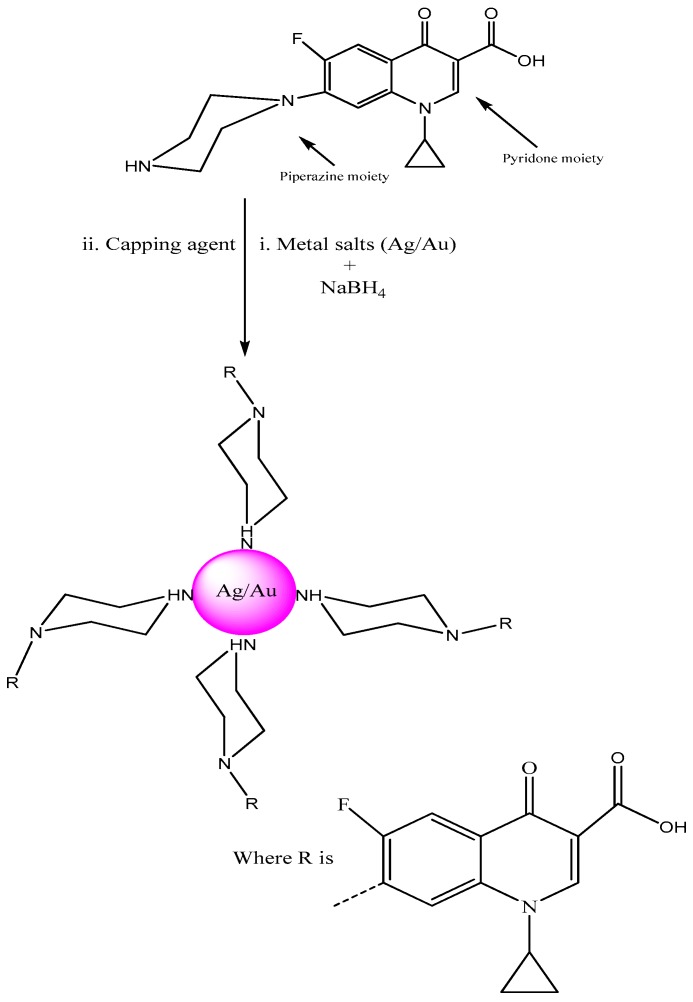
Stabilizing and capping action of ciprofloxacin with noble metals (Ag/Au).

The present manuscript proves the utility of ciprofloxacin as a stabilizing and capping agent for the synthesis of metallic (Ag/Au) NPs. The effect of salt (1 M NaCl), pH and temperature were studied. This fluoroquinolone drug was nominated due to the NH moiety in its structure which has the capability to cap Ag/Au NPs (Figure 1).

### 2.1. UV-Visible Spectroscopy and Optical Inspection of Ag/Au-cip NPs

The optical properties of Ag-cip and Au-cip NPs were determined by UV-Visble spectroscopy, the most frequently used method, to ascertain the synthesis and stability of metallic nanoparticles. The formation of Ag and Au NPs is evident from the presence of surface plasmon resonance (SPR) absorption bands from 400–500 nm and from 500–600 nm, respectively. Another method is to observe the color variation before and after the formation of nanoparticles. It was observed that the colorless solution became colored after addition of the reductant and indicating the formation of Ag/Au-cip nanoconjugates.

**Figure 2:**
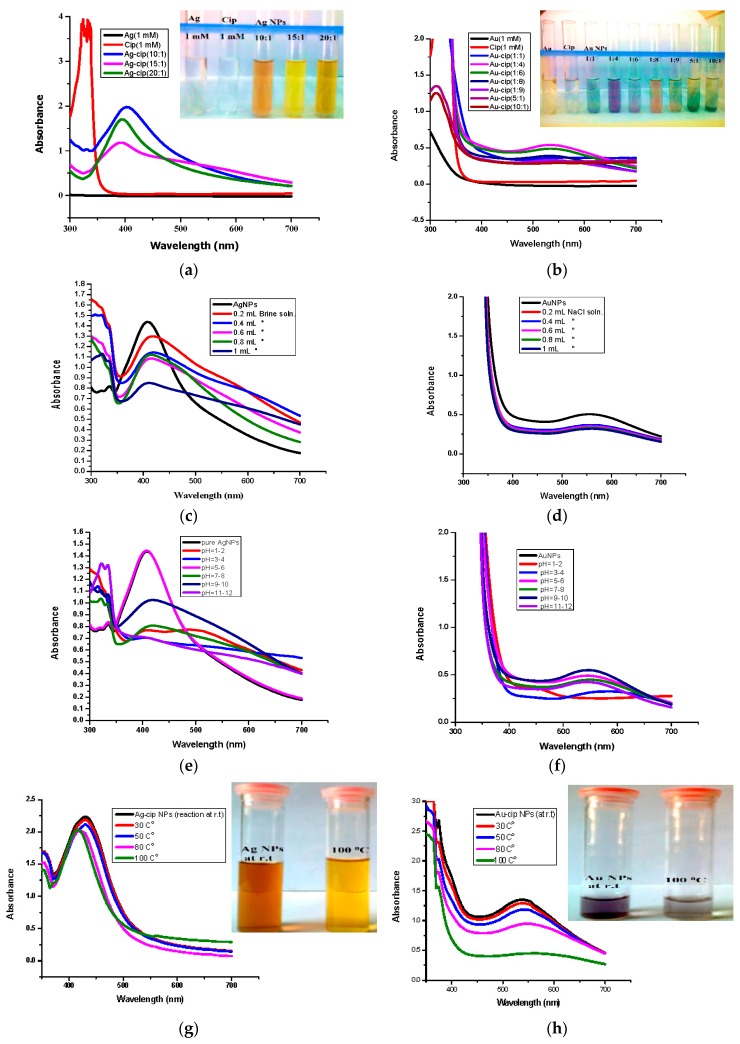
(**a**) Optimized UV-Vis spectra of Ag-cip NPs (Inset: Ag-cip NP color); (**b**) Optimized UV-Vis spectra of Au-cip NPs (Inset: Au-cip NP color); (c) Effect of salt on the stability of Ag-cip NPs; (**d**) Effect of brine on the stability of Au-cip NPs; (**e**) Effect of pH on the stability of Ag-cip; (**f**) pH effect on the stability of Au-cip; (**g**) Effect of temperature on the stability of Ag-cip NPs. (Inset: effect of temperature on Ag-cip NP color; (**h**) temperature effect on the stability of Au-cip NPs. (Inset: effect of temperature on Au-cip NP color).

To figure out the optimal conditions, reactions with numerous metal and ligand ratios were conducted; keeping the ligand (cip) as constant and varying the amount of metal (silver/gold), *i.e.*, (5:1, 10:1, 15:1, 20:1) and *vice versa i.e.*, (1:2, 1:4, 1:6, 1:8, 1:9). The ratios which showed the highest absorption peaks were explored further. The best peak for Ag-cip NPs was perceived for a reaction of 10:1 (metal: ligand), while for Au-cip NPs, the sharpest surface plasmon band was observed at 1:4 (metal: ligand) as shown in the Figure 2a,b, respectively. Figure 2c,d illustrate the effect of brine solution on the SPR peak of Ag/Au NPs. A greater concentration of sodium chloride decreases the absorbance maxima. This fast reduction in absorbance of Ag/Au NPs in the presence of NaCl is attributed to to the aggregation caused by Cl^–^ ions. This shows that aggregation dominates at increased salt concentrations. The stability of Ag/Au NPs was higher in water compared to salt solution.

For the pH study, 3 mL of Ag and Au-cip NPs was used. The pH of Ag-cip was 5.49, while that of Au-cip was 9.56.The pH of Ag/Au-cip (7–12) was adjusted by 1 M NaOH solution. Also, the pH of these metallic nanoparticles (1–7) was maintained by using 1 M HCl. The UV–Visible results were noted after 24 h (Figure 2e,f). Temperature is one of the important environmental factors that influence the stability and chemical characteristics of Ag/Au NPs. The temperature effect was also examined for these metallic NPs with the help of UV-Visible spectroscopy. Ag-cip NPs were stable under a greater variation in temperature, as shown in Figure 2g. The Surface Plasmon Resonance (SPR) bands up to 100 °C showed a slight decrease in intensity with a blue shift of the original band, which is attributed to the degradation of nanoparticles with a decrease in particle size. Similarly Au-cip NPs showed stability to increasing temperature up to 100 °C. Exploring the effect of temperature on the color of Au-cip NPs indicated that color of NPs changed from pinkish-purple to light blue, revealing the aggregation of Au NPs with the rise in temperature as shown in the inset of Figure 2h.

### 2.2. FTIR and AFM Studies

**Figure 3:**
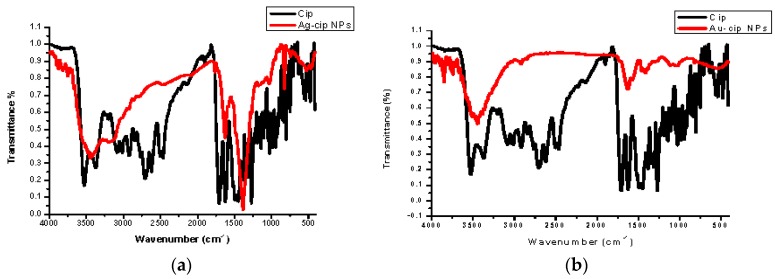

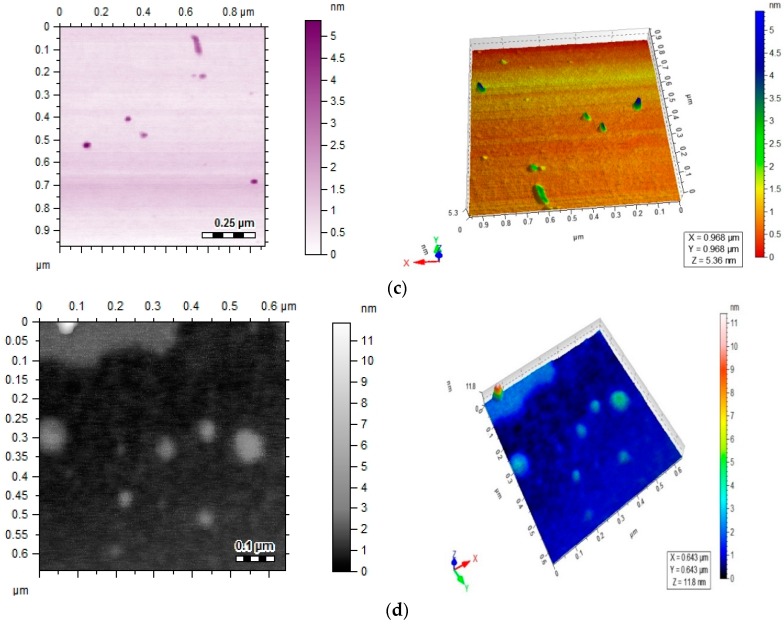
(**a**) FTIR spectra of Ag-cip NPs; (**b**) FTIR spectra of Au-cip NPs; (**c**) AFM image of silver nanoparticles; (**d**) AFM image of gold nanoparticles.

Spectral data was obtained by FTIR analysis for pure ciprofloxacin, Ag-cip and Au-cip NPs. Bands corresponding to C–H, N–H. C=O and OH stretching were observed, as well as OH twisting of the COOH group at 2918, 3372, 1708, 2706 and 1448 cm^−1^, respectively. Moreover, the band at 3528 cm^−1^ shows the O–H stretching of the carboxylic acid group in the pure drug. The peak at 3372 cm^−1^ is moved to 3448 cm^−1^ and broadened for Ag-cip NPs, while in case of Au-cip NPs the signal at 3372 cm^−^^1^ shifted to 3425 cm^−1^. This peak at 3372 cm^−1^ is due to the N–H stretching of the piperazine group that is considered to be responsible for stabilizing the Ag/Au-cip NPs. In the case of Ag NPs the FTIR spectral bands indicated a carbonyl peak shift from 1708 cm^−1^ to 1628 cm^−1^ and 1388 cm^−1^, whereas Au NPs showed a shift from 1708 cm^−1^ to 1626 cm^−1^ and 1384 cm^−1^ (Figure 3a,b). Surface morphology of the ciprofloxacin-capped Ag and Au NPs was studied by atomic force microscopy (AFM; Figure 3c,d). The AFM images show that the Ag-cip NP nanoconjugates are spherical in shape with sizes in the range of 40 to 50 nm, whereas the Au-cip NPs also possess spherical shape and shows calculated sizes from 60 to 85 nm.

### 2.3. EDX and SEM Studies

**Figure 4:**
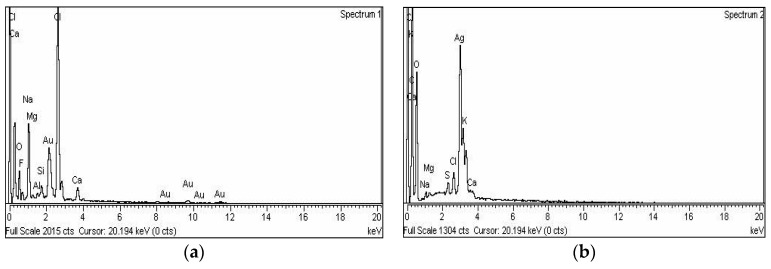

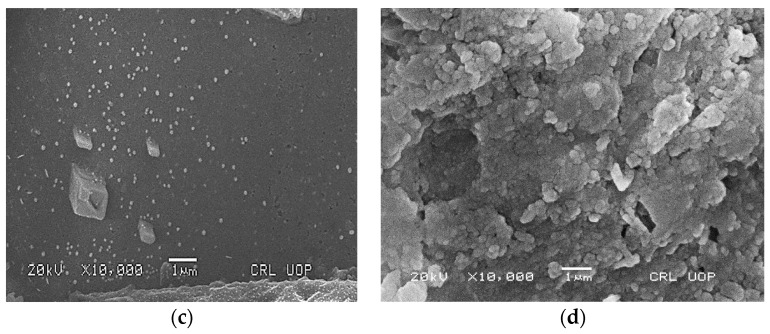
(**a**) EDX spectrum of Ag-cip NPs; (**b**) EDX spectrum of Au-cip NPs; (**c**) SEM image of Ag-cip NPs; (**d**) SEM image of Au-cip NPs.

Energy-dispersive X-ray spectroscopy demonstrated the elemental nature of the metallic nanoparticles using ciprofloxacin as stabilizing and capping agent. The appearance of a signal in the region of silver in the EDX analysis proves the synthesis of silver nanoparticles. Metallic Ag usually show an optical absorption peak at nearly 3 keV due to SPR. The peak for silver appeared at 3 keV. For Au-cip NPs, the EDX band indicates the existence of characteristic gold peaks at 2.12 and 9.71 keV as shown in Figure 4a,b. Scanning electron microscope (SEM) analysis confirmed that MNPs are nano-sized. The spherical shape of the Ag and Au NPs is shown in the SEM images (Figure 3c,d).

### 2.4. Urease Inhibitory Assay

The synthesized metallic nano-congugates (Ag-cip and Au-cip) along with the parent drug, ciprofloxacin (cip), were marked for jack bean urease enzyme inhibition studies to explore their inhibition potential. After screening of Ag, cip, Ag-cip and Au-cip against urease enzyme, Ag-cip was found to be significantly active with 96% inhibition at 0.2 mg/mL (Table 1). The Ag-cip NPs also showed significant activity with an IC_50_ value of 1.181 ± 0.02 μg/mL. The Au-cip NPs showed appreciable urease inhibitory activity with an IC_50_ value of 52.55 ± 2.3 μg/mL and 90% inhibition at 0.2 mg/mL as compared to the parent ciprofloxacin, which was found less active with 75% inhibition at 0.2 mg/mL and IC_50_ = 82.95±1.62 μg/mL. From all these analysis one can deduce that after capping ciprofloxacin with Ag and Au, the activity of ciprofloxacin was significantly enhanced (Table 1). Conjugation of cip to Ag and Au had a strong inhibition effect when compared with ciprofloxacin with no capping.

### 2.5. Antibacterial Activity

The ciprofloxacin and its stabilized metallic nanoparticles (Ag-cip and Au-cip NPs) were tested against *Bacillus subtilis*, *Klebsiella pneumoni* and *Staphylococcus aureus* (Table 2). The metallic nanoparticles revealed significant antibacterial activity against all three test stains. The Ag-cip NPs showed good antibacterial activity against the three strains with inhibitory zones from 20–24 mm at 3 mg/mL, whereas for the parent drug (cip) the zone of inhibition ranged from 24–28 mm. The Au-cip also exhibited significant antibacterial activity against the three strains with inhibition zones from 22–24 mm. Streptomycin was used as standard in the study.

## 3. Experimental Section

### 3.1. Materials and Chemicals

Sodium borohydride (NaBH_4_) was purchased from Wako Pure Chemical Industries (Tokyo, Japan). Silver nitrate (AgNO_3_) was obtained from Sigma-Aldrich (St. Louis, MO, USA) while hydrogen tetrachloroaurate (III) trihydrate (HAuCl_4_·3H_2_O), sodium carbonate (Na_2_CO_3_), hydrogen chloride (HCl), sodium hydroxide (NaOH), sodium chloride (NaCl) and methanol (CH_3_OH) were purchased from Merck (Kenilworth, NJ, USA). Ciprofloxacin was a kind gift from the BIOREX local Pharmaceutical Company (Islamabad, Pakistan). All chemicals in this assignment were analytical grade. Solutions were prepared in deionized water and stored in dark. Glassware was washed with aqua regia, then with distilled water and finally dried.

### 3.2. Synthesis of Gold and Silver Nanoparticles

Na_2_CO_3_ was used to neutralize ciprofloxacin HCl. This was done to prevent the production of AgCl precipitates during the NP synthesis. Ag-cip and Au-cip NPs were prepared with NaBH_4_ capped with ciprofloxacin (cip). Several reactions were conducted with changing volumes of ciprofloxacin (1 mM), 1 mM of AgNO_3_ and 1 mM of HAuCl_4_. The reaction mixtures were stirred strongly for 30 min and 0.2 mL of 50 mM NaBH_4_ was gradually added. The stirring was done for 30 min. The color variations indicated formation of Ag and Au-cip NPs. When the reducing agent was added, the bright yellow solution changed to different colors. The ratios with the sharpest absorption peaks were subjected to further investigation. The formation of Ag and Au-cip nanoconjugates was further confirmed by UV-Visible spectroscopy, AFM and FTIR. Bioactivity was evaluated for Ag/Au-cip nanoparticles having the finest size and morphology.

### 3.3. Factors Affecting the Stability of Silver and Gold Nanoparticles

#### 3.3.1. Salt Effect

Stability of Ag and Au NPs were also examined against NaCl solution (1 M). Afterwards, UV-Visible spectra were obtained. Deviation or persistence of the UV-Visible peaks provided strong proof for the stability of MNPs.

#### 3.3.2. pH Effect

The pH of the reactions were recorded at pH values 2–13 by using 1 M HCl and 1 M NaOH. The absorption of these solutions was measured spectrophotometrically.

#### 3.3.3. Temperature Effect

The temperature effects on the synthesized Ag-cip and Au-cip NPs were studied by taking 5 mL of freshly prepared colloidal NPs in 25 mL flasks. Heating of the solutions was done for 30 min up to 100 °C. The nanoparticles were then kept at room temperature and their UV-Visible spectra were noted.

### 3.4. Characterization Techniques

#### 3.4.1. UV-Visible Spectroscopy

The UV-Visible spectra of the metallic NPs were carried out with a Shimadzu UV-240 spectrometer (Hitachi, Japan) in the range of 300 to 700 nm. The spectra of Ag and Au NPs are the initial signal of synthesized Ag/Au NPs. The presence of specific absorption peaks, 500–600 nm and 400–500 nm, deep-rooted the synthesis of gold and silver NPs, respectively.

#### 3.4.2. Fourier Transform Infrared Spectroscopy (FTIR)

This analysis was conducted with a Shimadzu IR-460 spectrophotometer. For this measurement the capped metallic NPs were freeze dried and a small quantity of lyophilized sample (0.01 g Ag/Au NPs) was blended with KBr for FTIR analysis.

#### 3.4.3. Atomic Force Microscopy (AFM)

The shapes and sizes of the nanomedicines were characterized by atomic force microscopy, using a Multimode Nanoscope IIIa instrument (Veeco, Santa Barbara, CA, USA) in tapping mode. For this determination, the Ag and Au-cip NPs samples were prepared by dissolving thin films in triply distilled water and scattering on a cleaved sheet of mica. The atomic force microscopic images were recorded at ambient temperature and repeated with different concentrations of the samples.

#### 3.4.4. Scanning Electron Microscopy (SEM) and Energy Dispersive X-ray (EDX) Analysis

Synthesized MNPs were also characterized by using SEM and EDX techniques. These analyses were performed with a JSM591 SEM with EDX instrument (JEOL, Tokyo, Japan). EDX demonstrated the chemical nature of these NPs.

### 3.5. Urease Inhibitory Assay

The synthesized metallic nano-conjugates (Ag/Au-cip) were subjected to urease inhibition activity assays. Reaction blends containing jack bean (*Canavalia ensiformis*) urease (25 μL), 100 mM of urea, 55 μL of buffer (pH 6.8) and 5 μL of different concentrations of test samples (0.5 to 0.00625 mM) were incubated in 96-well plates at a temperature of 30 °C for 15 min. In the kinetics studies, numerous concentrations of substrates and test compounds were used. Afterward 45 μL of phenol reagent (1% *w*/*v* phenol and 0.005% *w*/*v* sodium nitroprussside), and 70 μL of alkali (0.5% *w*/*v* NaOH and 0.1% *w*/*v* NaOCl) were added to separate wells. After 50 min, the cumulative absorbance (630 nm) was measured in triplicate in a microplate reader (SpectraMax M2, Molecular Devices, Sunnyvale, CA, USA). The production of NH_3_ was measured by using the indophenol technique with a standard (thiourea) [37]. Ultimately the results were analyzed by the SoftMax Pro software (Molecular Devices, Sunnyvale, California, USA), MS-Excel (Microsoft, Remond, WA, USA) and Ez-fit (Souderton, PA, USA) programs. The following formula was used for calculating percent inhibition:

% Inhibition = 100 − (OD test/OD control) × 100

### 3.6. Antibacterial Activity

The antibacterial assays were performed by the well diffusion method using Mueller Hinton agar. The cultures were developed in triplicate at 37 °C for 72 h. The broth values (0.6 mL) of the organism was placed in a sterile Petri-dish then 20 mL of the sterile molten Mueller Hinton Broth (MHB) was added. Streptomycin was used as a standard (2 mg/mL). Inoculation was performed for one hour for diffusion of the antimicrobial agent into the medium. After incubation (37 °C for 24 h), the widths of the microbial development inhibition zone were measured in millimeters (mm). The tests were repeated in triplicate.

## 4. Conclusions

A robust and effective procedure employing NaBH_4_ as a mild reducing agent to yield silver and gold nanoparticles stabilized with ciprofloxacin was introduced. Spectroscopic studies revealed that the NH moiety of the cip piperazine group is mainly responsible for the capping and stabilization of these metallic nanoparticles. These nano-conjugates exhibited excellent stability against differences in brine solution concentration, pH and heat. The energy-dispersive X-ray spectroscopy analysis showed the inorganic composition of the synthesized Ag/Au NPs. The synthesized MNPs are readily water soluble and safe. They were found to be efficient selective urease inhibitors in comparison to Ag and cip and also indicated significant antibacterial activity. Further exploration of the biological potential of the presented metallic MNPs is under investigation in our research laboratory.

## Figures and Tables

**Table 1:** Urease enzymes inhibition studies of Ag, ciprofloxacin, and ciprofloxacin-capped Ag/Au nanoparticles.

Sample	Ag	Ciprofloxacin	Ag-Cip NPs	Au-Cip NPs	STD
% Inhibition	29.2	75	96	90	98.2
Concentration (mg/mL)	0.2	0.2	0.2	0.2	0.5 (mM)
IC50 ± S.E.M (μg/mL)	NA	82.95 ± 1.62	1.181 ± 0.02	52.55 ± 2.3	21 ± 0.11 (µM)

S.E.M = Standard error mean; STD = Standard; Cip = ciprofloxacin.

**Table 2:** Antibacterial activities of ciprofloxacin and its metallic (Ag/Au) nanoparticles (zone of inhibition (mm) at 3 mg/mL).

Bacterial Strain	Strain	Ciprofloxacin	Ag-Cip NPs	Au-Cip NPs	Streptomycin (STD)
*S. aureus*	+	26	24	22	26
*Bacillus subtilis*	+	28	22	24	26
*K. pneumoniae*	-	24	20	24	28

Well size = 6 mm, STD = Standard.
